# A Metrological Near-Room-Temperature Photon-Number-Resolving Detector: A Design Study

**DOI:** 10.3390/s25175470

**Published:** 2025-09-03

**Authors:** Zachary H. Levine, Joshua C. Bienfang, Alan L. Migdall, Neil M. Zimmerman

**Affiliations:** 1 Quantum Measurement Division, National Institute of Standards and Technology, Gaithersburg, MD 20899-8410, USA; joshua.bienfang@nist.gov (J.C.B.); migdall@nist.gov (A.L.M.); 2 Joint Quantum Institute, College Park, MD 20742-0001, USA; 3 Nanoscale Device Characterization Division, National Institute of Standards and Technology, Gaithersburg, MD 20899-8423, USA; neil.zimmerman@nist.gov

**Keywords:** photon counting, room-temperature silicon device, low error rate

## Abstract

We describe and model a non-cryogenic optical detector designed to count incident photons with metrological accuracy. Our design consists of a semiconductor device operating at −10 °C and is predicted to resolve pulses of up to 10 photons with an error rate of 2% in the input number of photons. We present an estimate of the overall device performance using a combination of estimates and simulations of optical loss, discrete electron loss and noise, and electronic noise.

## 1. Introduction

An optical detector with metrological-grade photon-number error rate (i.e., a high probability that the detector output correctly reports the number of photons incident on its input) not only represents a primary standard for optical-power calibration, but also provides a critical enabling technology for photonic quantum information science [1,2,3,4,5,6]. Unfortunately, achieving low photon-number error rate in real-world devices is uniquely challenging because it requires both low noise and high single-photon system detection efficiency, as well as high photon-number resolution. These challenges only increase as the photon number increases. For example, a device with single-photon detection efficiency of 99% (1% error rate) has a 10 photon error rate of nearly 10%. Typically, the technologies that provide the highest single-photon detection efficiency (typically, 99%) have required cryogenic cooling, to 1 K or lower. On the other hand, detectors that operate near room temperature do not reach the highest detection efficiencies or either do not provide clear photon-number resolution [7] or provide very limited photon-number resolution [8,9].

The ability to detect single photons does not necessarily imply the ability to have a photon-number resolving (PNR) device. For example, consider single-photon avalanche detectors (SPADs) and microchannel plates. For such systems, although single photons can be detected, the noise inherent in the first step of the avalanche process—it could yield two or three or more electrons—means that these have not been candidates as PNR detectors to date, at least not as single devices.

As a result, one common method to achieve some degree of photon-number resolution is to multiplex non-PNR detectors to form a quasi-PNR detector [10,11]. Unfortunately, this approach is inherently limited by the fact that there is always a non-zero probability that multiple photons will end up at the same non-PNR detector, and the fact that the noise from each individual detector (or time bin, for time-division multiplexing) is additive in the overall output. The combination of these two effects with real-world devices means that the photon-number error rate is rather poor in limited-PNR and quasi-PNR detectors [12] for more than just a few photons.

One exception is the transition edge sensor (TES), a microcalorimeter made of a thin metallic absorber held at its superconducting-to-normal transition, typically near 100 mK, in which the energy of one photon (or more) causes a measurable change in resistance [13]. Not only can these detectors have extremely low noise [14], they can also have high detection efficiency and excellent photon-number resolution, meeting all the requirements for low photon-number error rate; a TES in an integrated optical cavity with carefully designed fiber coupling demonstrated single-photon detection efficiency as high as 0.98±0.01 (Ref. [15]), and another TES was able to observe up to 29 photons at 1570 nm [16]. Thus, in spite of their extreme operating temperature and the attendant cost, size, and power requirements, TESs are the detector of choice for low photon-number error rate and they serve as a critical enabling technology for some photonic quantum processors [6].

There have been other efforts to develop room-temperature PNR detectors using microelectronics technology, primarily focused on detecting single charge carriers with nanoscale field-effect transistors [17,18,19]. The steady advance in complimentary metal-oxide semiconductor (CMOS) fabrication technologies and CMOS cameras has now achieved imaging arrays in which some pixels exhibit root-mean-square (RMS) readout noise much lower than 0.5 *e*, that is, individual photo-generated charge carriers can be resolved, even at room temperature [20]. This increased charge sensitivity arises from the extremely low capacitance (<1 fF) achieved at the gate of the readout transistor [21], which results in a conductance change that is large enough to discriminate (with sufficient averaging and noise suppression) photoelectron numbers at the transistor gate. A study in 2017 showed that in a single particularly high-resolution pixel, or “jot,” in a megapixel array, photon-number resolution was observed [22]. A noteworthy aspect of this approach is that its mode of operation puts few restrictions on the physical geometry of the optical absorption region. This suggests that this approach may be compatible with a metrological-grade detector in which the active area and optical depth of the absorption region is optimized for near-unity absorption at some wavelength and photocarrier loss is low, raising the tantalizing prospect of developing a detector that operates at room temperature and provides metrological grade photon-number error rate for pulsed sources.

Here we explore this possibility with a numerical study that pays particular attention to tabulating the accumulated error rate in photon number due to imperfections and processes that would occur in a real-world device. We consider a large-area single-pixel silicon device with a deep photo-absorption region, and we focus on the device’s ability to gather photo-generated carriers from the absorption region with low loss and collect them at an ultra-low-capacitance readout gate. Our model reports that 10 visible light photons (400 nm ≤λ≤700 nm) can be resolved with an error rate in the photon number of 2.1%, and identifies optical coupling efficiency, carrier recombination, and background carrier generation as the dominant contributors to the error rate. (See Appendix A for a brief discussion of related terms and Appendix B for the mathematical framework.) This study suggests that a metrological-grade PNR detector near room temperature may be feasible, and serves to motivate the further investigation of such devices.

It is useful to emphasize the difference between our goal—extremely high quantum efficiency in a single-pixel detector—and that of other areas such as astronomy where 50% to 95% quantum efficiency in a megapixel array is the goal [23,24].

## 2. Design and Performance

### 2.1. System Overview

**Figure 1:**
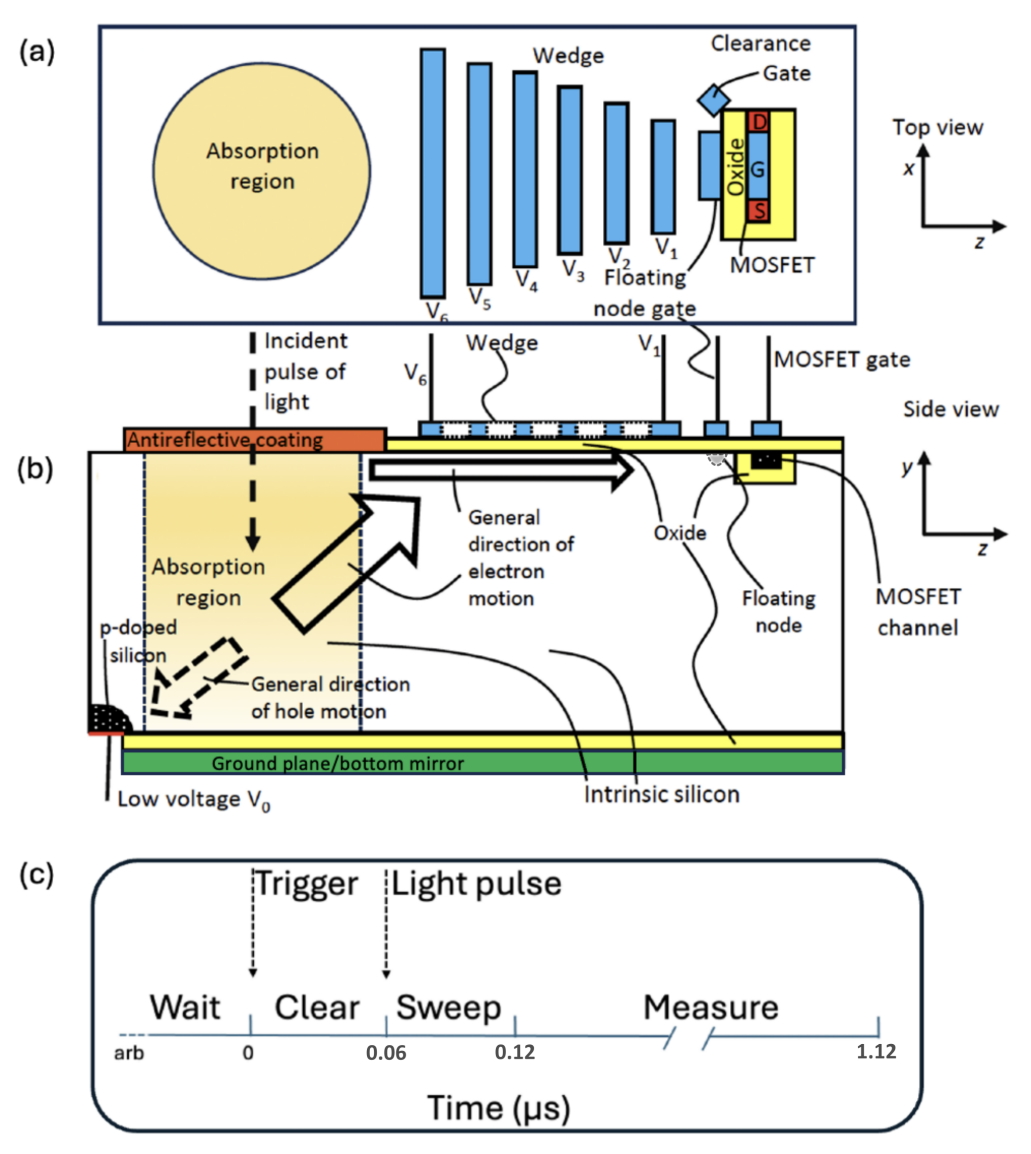
Device (not to scale) (**a**) top view, and (**b**) side view, with (**c**) timing diagram. A pulse of light enters the device at near-normal incidence through an antireflective coating into a cylindrical absorption region, which is intrinsic silicon. An electric field separates electrons and holes. The holes exit the device through an ohmic contact protected by a heavily p-doped region. The electrons travel to the upper right toward the wedge and then along the surface to the right into the floating node. The wedge electrodes obey $V_{6}<V_{5}<\cdots <V_{2}<V_{1}$. Although six individual electrodes are shown, the actual device will have many electrodes. (For brevity, we refer to these electrodes as “wires” in the following.) The electrons in the floating node influence the current on a short-channel MOSFET which is on the opposite side of a trench oxide [25]. The absorption region is shown in peach, with the antireflective coating at the entrance face shown in orange. The gate electrode are shown in blue except the ground plane which is shown in green. Ohmic contacts are are shown in red. The oxide layers are shown in yellow. The p-doped regions are shown in black with white dots. Source, gate, and drain are indicated by S, G, and D. (**c**) After waiting for an arbitrary time (including zero), a trigger pulse initiates clearing electrons from the device. The light pulse should arrive 0.06 μs after the trigger, and electrons are swept to the floating node. The measurement phase for the electrons extends from 0.12 μs to 1.12 μs.

We begin with a brief overview of the operation of the device illustrated in Figure 1. Photons enter at near-normal incidence through an anti-reflection coating into the cylindrical absorption region, which is composed of intrinsic silicon and defined physically by the entrance facet and the bottom of the device, but is not confined laterally. A reflective surface on the back of the device recycles any photons that were not absorbed back through the absorption region. In addition, an external mirror serves a similar purpose. The device performance is relative to ideally counting the number of photons which arrive at the entrance surface.

Photoabsorption generates electron–hole pairs. (In this work, we use the term “electrons” to mean conduction-band electrons.) The electrons and holes are pulled in opposite directions by a static, diagonal electric field created between a low-voltage p-doped region and a higher-voltage “wedge” electrode on the top of the device, as illustrated in Figure 1. The holes are continuously cleared by passing through the p-doped region to an ohmic contact on the bottom of the device. The p-doped region adjacent to intrinsic silicon functions like a pn-diode, allowing a hole current to the ohmic contact, but not an electron current.

The electrons drift up the electric field to the top of the device to the right of the absorption region. There, they are collected by electrodes arranged in a wedge pattern (see Figure 1) and continue traveling to the right near the silicon surface under the influence of successively higher voltage electrodes until they occupy a region of the silicon which we refer to as the “floating node.” The electrons in the floating node influence the current in a short-channel metal-oxide-semiconductor field-effect transistor (MOSFET). The number of electrons on the floating node are found by analyzing this current. The device is examined in detail in the rest of this section. We envision the fabrication of this device involving processing on both the top and bottom sides. Device parameters are given for 300 K and 263 K (−10 °C) operation, well within the range of small thermoelectric coolers referenced to room temperature. We show in Section 2.6 that the lower temperature is necessary to achieve a low rate of thermally-generated carriers.

### 2.2. Photoabsorption Region

Antireflective coating and external mirror. The photoabsorption region is a cylindrical region with an anti-reflective (AR) coating on the entrance port and a mirror under a thin oxide layer which is the “ground plane” in Figure 1b. We define the device efficiency relative to photons arriving at the front surface of the AR coated window. To avoid condensation a window is required. The passage of photons through the AR coating represents the first source of error.

This design leads to three surfaces which can reflect light, namely the front surface of the room temperature window, the back surface of this window, and the front surface of the absorption region. For the purpose of estimation, we assume the reflectivity from each surface is 1%, which is easily commercially available. Cumulatively, we have an upper bound of 3% reflectivity from these three surfaces.

**Figure 2:**
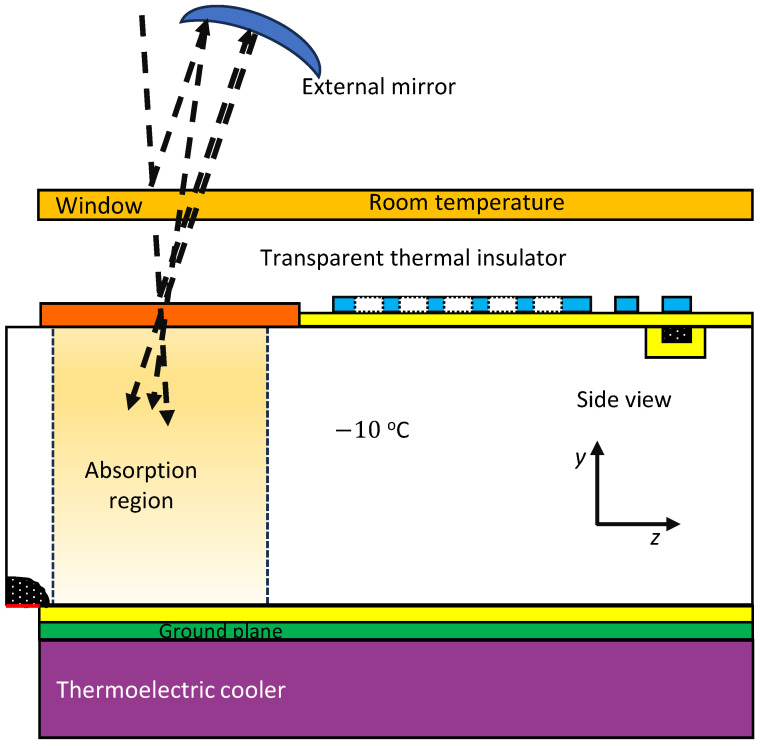
The device requires some discrete components which shown here along with the integrated portion of the device shown in Figure 1b. The new features are a thermoelectric cooler bonded to the silicon, and a curved, external mirror to reflect the small amount of light reflected from various surfaces on incidence. The dashed lines show the main optical path and two of the more prominent reflections. The effect of Snell’s law—the bending of light toward the normal in the higher index medium, namely silicon—is not shown to keep the figure readable.

If there were 10 incident photons in a pulse, then on average 0.3 photons will be reflected. This figure is too high for our design goal of an error rate of 2% or less. To address that, one option would be to pursue higher performance AR coatings which could bring the reflectivity per surface down to as low as 0.1% [26]. However, three such surfaces would still reflect 0.03 photons on average from 10 incident photons which is still not consistent with our design goal. Hence we opt to add an external mirror with a reflectivity of at least 99.4%, as shown in Figure 2. Such mirrors are commercially available. The external mirror radius of curvature matches the distance from the absorption region to the mirror so as to direct reflected photons back to the detector. Considering only single reflections, the fraction of photons entering the system rises to 0.9998. Continuing with our example with 10 incident photons, on average 0.002 photons would not enter the absorption region.

Aperture size. In addition to reflections off the input surface, discussed above, some of the light may land outside the finite aperture of the device and be lost to absorption or reflection.

The power of a TEM_00_ Gaussian beam passing through an aperture with radius *a* is given within geometrical optics by

(1) $$\begin{array}{c} \frac{P}{P_{0}}=1-\exp\left(-\frac{2a^{2}}{w_{0}^{2}}\right) \end{array}$$

where $P_{0}$ in the incident power and *P* is the transmitted power, and $w_{0}$ is the Gaussian beam waist parameter. For metrological applications we want $P/P_{0}\geq 0.999$. We select the diameter of the aperture to be 125 μm, a compromise between a size large enough to facilitate low-loss optical coupling and small enough to ensure the efficient collection of photo-generated electrons from the absorption region with a small number of dark counts; thus, a=62.5 μm. To achieve $P/P_{0}=0.999$, Equation (1) gives $w_{0}=18$ μm. Coupling with this efficiency has been demonstrated experimentally at 851 nm into a 150 nm [27]. The ratio of λ/a is nearly equal between this experiment and our proposed device. As an example, for the wavelengths λ=400 nm and λ=700 nm the numerical aperture (NA) is given by $\sin[\lambda /\left(\pi w_{0}\right)]=0.007$ or 0.012 respectively. Such a low values of NA are easily implemented. The Rayleigh length $z_{R}$ is given by

(2) $$\begin{array}{c} z_{R}=\frac{\pi w_{0}^{2}}{\lambda }. \end{array}$$

For our example, $z_{R}=2.5$ mm for λ=400 nm and $z_{R}=1.4$ mm for λ=700 nm, which is much longer than the optical path in the device, which is at most four times 30 μm, i.e., 120 μm. This Rayleigh length is much larger than the device so the beam may be taken to be collimated.

Absorption. We now estimate the fraction of the light that gets absorbed by the device. That fraction will ultimately limit the efficiency and error rate of our detector. The absorption length of light in silicon decreases with wavelength within the visible [28], so we will consider λ=700 nm, our worst case. For reference, the indirect bandgap of silicon is 1.12 eV or λ=1100 nm. The thickness of the device is chosen to be 30 μm. The presence of a mirror on the bottom face doubles the available thickness although there is some absorption loss within the mirror. Such losses will decrease with increasing wavelength which is fortunate as the mirror’s performance gets better as we rely on it more heavily. (See, e.g., the reflectance curve for silver mirrors in Figure 4 of Ref. [29].) The absorption in two passes through the absorption region is given by

(3) $$\begin{array}{c} A\left(\lambda \right)=\left[1-\exp\left(-\alpha \left(\lambda \right)L\right)\right]\left[1+R_{M}\left(\lambda \right)\exp\left(-\alpha \left(\lambda \right)L\right)\right] \end{array}$$

where *L* is the thickness of the absorption region, $R_{M}\left(\lambda \right)$ is the reflectivity of the mirror and α(λ) is the absorption coefficient in silicon. Both are wavelength-dependent. The second reflection off of the antireflection coating is neglected. The analysis is continued in Appendix C.

For λ=700 nm, the absorption length is 5.2 μm. Passing through 30 μm of silicon leads to an absorption of 0.9969. Assuming that we can coat the silica with a 97% reflectivity mirror, an additional (0.0031)(0.97)(0.9969) = 0.0030 of the light will be absorbed in a return pass, for a total absorption of 0.9999 of the incident light. In principle, the external mirror provides for a third pass through the system, but the light is sufficiently weak that we may neglect this. Our estimate of 97% reflective mirrors at 700 nm is based on the commercial availability of such mirrors made with silver on silica across the visible. Moreover, a failure to achieve this parameter could be mitigated by a small increase in the silicon thickness, e.g., from 30 μm to 35 μm.

Generation of multiple photoelectrons. For a photon energy greater than twice the indirect band gap, namely 2.24 eV or 554 nm, it becomes possible to generate two photoelectron pairs from a single photon. Measurements, though, indicate that there is only a single photoelectron generated for photon energies up to 3 eV [30]. On the other hand, multiple carrier generation is significant below 370 nm (above 3.25 eV) [30]. The reference suggests that multiple photoelectrons are generated in large numbers starting at three times the bandgap, or 3.36 eV, for then a photoelectron carries enough energy to generate an electron–hole pair by impact ionization. Thus the lower bound of linear operation for the device can safely be taken to be 550 nm although it is likely to be closer to 400 nm. Thus we limit our device operation to the visible.

### 2.3. Electron Transport to Floating Node

The photoelectrons are created throughout the absorption region. They are attracted by a diagonal electric field created by the wedged electrode. Their motion can either be through the bulk or on the upper surface where their motion is confined by an oxide layer. Appendix D shows that if an electron obeys the drift equation with a constant mobility, and if there is a fixed voltage available to transport the electron from an initial position to a final position, then the shortest transport time will ultimately be the path with a constant electric field. The widely used method for moving electrons from pixel to pixel by clocking voltages as is done in charge-coupled devices (CCDs) [31] is not used here because the electrons can be generated in any part of the absorption region, and hence need different amounts of time to be collected. Our device differs from CCDs principally in the transport of electrons. The generation of carriers through photoabsorption and the use of readout electronics is analogous.

Holes are also generated in the photoabsorption process. Since the hole mobility is about three times less than the electron mobility, we will measure the number of photoelectrons and simply remove the holes. The holes are continuously swept away from the electrons to an ohmic contact held at low voltage, such as −1 V below ground, shown in the lower left corner of Figure 1b. There is a p-doped region with substantial doping such as an acceptor density of $10^{16}$ cm^−3^ next to the contact to prevent electrons from entering the device.

Entropy provides one bound on the minimum voltage we need to have a chance of moving the electrons from the absorption region to the floating node. The absorption region has a volume $V_{\mathrm{abs}}\approx 3.7\times 10^{-13}$ m^3^. As discussed in Section 2.4, the floating node has a volume of approximately $V_{\mathrm{FN}}\approx 10^{-24}$ m^3^. Hence, we must compress the electrons into $2.7\times 10^{-12}$ of the original volume. Assuming the number of microstates available to the electrons is proportional to the volume, we need a voltage difference of greater than $V_{\min}$ which obeys

(4) $$\begin{array}{c} \frac{V_{\mathrm{FN}}}{V_{\mathrm{abs}}}=\exp\left(-\frac{eV_{\min}}{k_{B}T}\right), \end{array}$$

where $k_{B}$ is the Boltzmann constant, *T* is the temperature, and *e* is the elementary charge. For temperatures of 300 K and 263 K, $V_{\min}$ is 0.69 V and 0.60 V respectively.

Equation (4) gives the voltage at which half of the electrons will be on the floating node. For our design, we require no more than a fraction η of the electrons to be off the floating node. The requirement becomes

(5) $$\begin{array}{c} \frac{\eta }{1-\eta }\frac{V_{\mathrm{FN}}}{V_{\mathrm{abs}}}=\exp\left(-\frac{eV_{\eta }}{k_{B}T}\right) \end{array}$$

For our design, $\eta_{0}=10^{-4}$ is a reasonable choice, leading to required voltages of $V_{\eta_{0}}$ to be 0.99 V for 300 K and 0.86 V. It is routine to have such voltages in a semiconductor device, so entropic considerations are not limiting.

A simple estimate of the time to move the electrons from the absorption region to the floating node is as follows. The electrons must travel through the bulk silicon comprising the absorption region for up to 125 μm. Next, the electrons must travel near the silicon-oxide interface under the wedge electrode for a little over half of that distance, say up to 75 μm, or a total of 200 μm. Earlier, we suggested that the electric field *E* should be constant. However, in the presence of a position-dependent mobility, the more exact condition is that the product μE should be constant. Using the argument in Appendix D and a 3:1 ratio of bulk mobility to surface mobility, 5/14 of the voltage drop should be in the 125 μm bulk transport section and 9/14 should be in the 75 μm surface transport section. Assuming 20 V applied voltage, an electric field of 57 kV/m is applied to the bulk section and 171 kV/m is applied along the surface. The electron drift velocity is given in Equation (A7). An estimate of the time it takes for the electrons to go from the absorption region to the floating node is derived from Table 1. The results are 36 ns at 300 K and 30 ns at 263 K. We double these to account for nonuniformity of the electric field and the additional time to clear due to diffusion. Although the 20 V may seem high compared to the standard practices in semiconductor electronics, the Skipper CCD, a photon-counting detector, is operated at 25 V [32].

In addition to drift, diffusion plays a role in modeling the motion of electrons in a semiconductor. The diffusion coefficient *D* is related to the mobility by the Einstein relation

(6) $$\begin{array}{c} D=\mu \frac{k_{B}T}{e}. \end{array}$$

Relevant values are given in Table 1. The root mean square (RMS) displacement ${\langle r^{2}\rangle }^{1/2}={\left(6Dt\right)}^{1/2}$, where *r* is the distance a particle has diffused in time *t*. At 300 K our time estimate is 72 ns and ${\langle r^{2}\rangle }^{1/2}=41$ μm. With 60 ns at 263 K, the figure is also 41 μm. Since this figure is comparable to but smaller than the 125 μm size of the absorption region, diffusion plays a role in the device simulation, but not a dominant role. Technically, we should reduce the diffusion coefficient at the silicon-oxide interface since the mobility is lower, but we omit this refinement to simplify the argument. If the lower surface diffusion were included, the required time would be a few percent smaller.

We may refine the estimate of the time to clear the electrons by including the effect of diffusion. During the time that the electrons are moving toward the floating node, they also spread out. Some have moved away from the floating node (“stragglers”) and need additional time to reach it. We can neglect lateral diffusion, as this does not increase the distance to the floating node by very much. As a 1D problem, the RMS displacement is given by ${\langle r^{2}\rangle }^{1/2}={\left(2Dt\right)}^{1/2}$, which differs from the previous value by a factor of $\sqrt{3}$. The time to clear the electrons is then given by

(7) $$\begin{array}{c} t=\frac{\ell_{0}+Z{\left(2Dt\right)}^{1/2}}{v} \end{array}$$

where $\ell_{0}$ is the maximum distance to travel, *Z* is a constant associated with how many standard deviations of the distribution we wish to retain, and *v* is the drift velocity. Equation (7) is a quadratic equation in $\sqrt{t}$, so taking $\ell_{0}=200$ μm, Z=4.265, and *v* and *D* from the 263 K values in Table 1, we find the time to clear the electrons is 43 ns vs. 30 ns without considering diffusion. (The *Z* value ensures all but $10^{-5}$ of the electrons will reach the floating node.) For our system estimate, we increase the value to 72 ns or 60 ns to allow for non-ideal behavior such as a non-uniform electric field as well as the diffusion correction. The duration of the pulse needs to be small compared to the transport time of 60 ns. This is commonly achieved in commercially available lasers.

In order to bring the photoelectrons from the absorption region, we place a series of electrodes of decreasing length from near the absorption region to near the floating node. These electrodes form a wedge next to the aperture and is an interface to the floating node as shown in Figure 1. The wedge is held at a positive voltage, increasing farther from the absorption region. Without the wedge structure, the floating node electrode takes much longer to attract the electrons of the absorption region. Combined with the ground below the absorption region, isolated by an oxide, and the ohmic contact at a small negative voltage used to drain holes, both shown in Figure 1, there is a diagonal electric field which sweeps the electrons out of the absorption region and to the wedge. Transport can occur either through the bulk silicon or along the surface of the silicon below the wedge.

**Figure 3:**
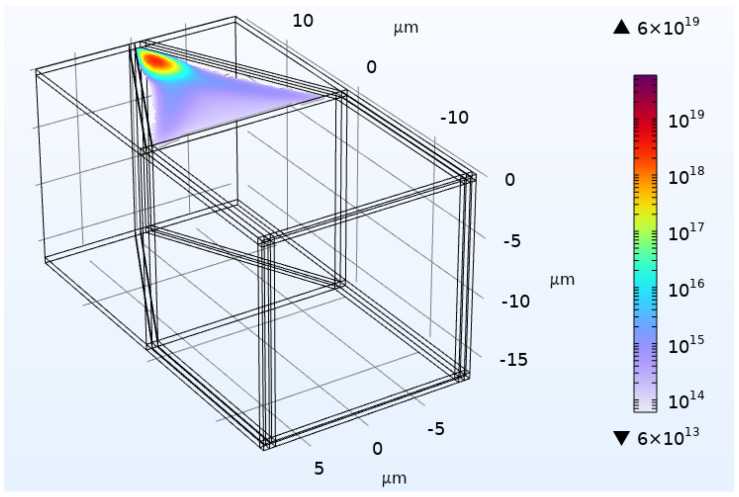
The final frame from a movie showing the transport of an electron cloud from the absorption region toward the floating node. The movie wedge22-01.avi is available in the Supplementary Materials (Video S1). At the start, the electrons are in the now-empty cube on the front, right side of the image. The final frame is for 5 ns. The simulation region is 32 μm×16μm×16μm. There is a ground plane at 0 V, a small electrode with −1 V at the lower right, and the wedge electrode goes from 2.5 V to 5.0 V. The color scale covers from $6\times 10^{7}$ cm^−3^ to $6\times 10^{13}$ cm^−3^. (The scale is given in units of m^−3^).

An example of the motion of the electrons is shown as a movie in Figure 3, calculated for a smaller version of the proposed device. The electrons move toward the wedge at first through the bulk but later along the surface. The simulation region is scaled down in order to simplify the computation. Also, the final transport to the floating node was done in a separate simulation not shown here. It takes less than 0.3 ns to evolve from a situation similar to the endpoint in the movie to the electrons being in the floating node. Because the floating node is so small, we found it too difficult to simulate the full absorption region and the floating node in one simulation. To get the electrons into the floating note after they have accumulated at the end of the wedge, the voltage on the wedge is lowered while keeping the floating node gate to a high voltage. The floating node voltage need not be as high as 20 V during the measurement period as long as it stays above the highest voltage on the wedge $V_{1}$.

Although we simulate a wedge, we envision the wedge to be made of individual electrodes. It is not clear how to best achieve a uniform electric field if the wedge were an unpatterned piece of metal even if a current were to flow through it. The individual electrodes composing the wedge may be controlled by individual external voltages, or they may be linked together by a thin wire with a current flowing through it. The voltage drop by Ohm’s Law gives a near-uniform electric field favored by the least-time theorem of Appendix D.

The movie ends with the electrons near the end of the wedge. Modeling with striped wedges indicates that with a 4 V higher potential on the floating node gate, 95% of the electrons appear directly below the floating node gate with only 0.003% of the electrons as far as one striped gate away (i.e., 100 nm with the 50 nm design rule used in this part of the simulation) within 0.3 ns. The two sections were modeled independently because of numerical problems associated with the three orders of magnitude different in length scales between the absorption region and the floating node.

A good design is to have 25 nm wide wires and spaces with the length of each wire decreasing by 200 nm and a gate oxide thickness of 20 nm. See Appendix E for a discussion of the oxide thickness and wire spacing.

### 2.4. MOSFET and Floating Node

The purpose of bringing the electrons to the floating node is so that they influence the current in the nearby MOSFET. The geometry of the floating node and the MOSFET is shown in Figure 1. We propose the wires, spaces, and oxide width to be 25 nm, as in the wedge. Both the gate and the floating node are taken to be 200 nm long.

**Figure 4:**
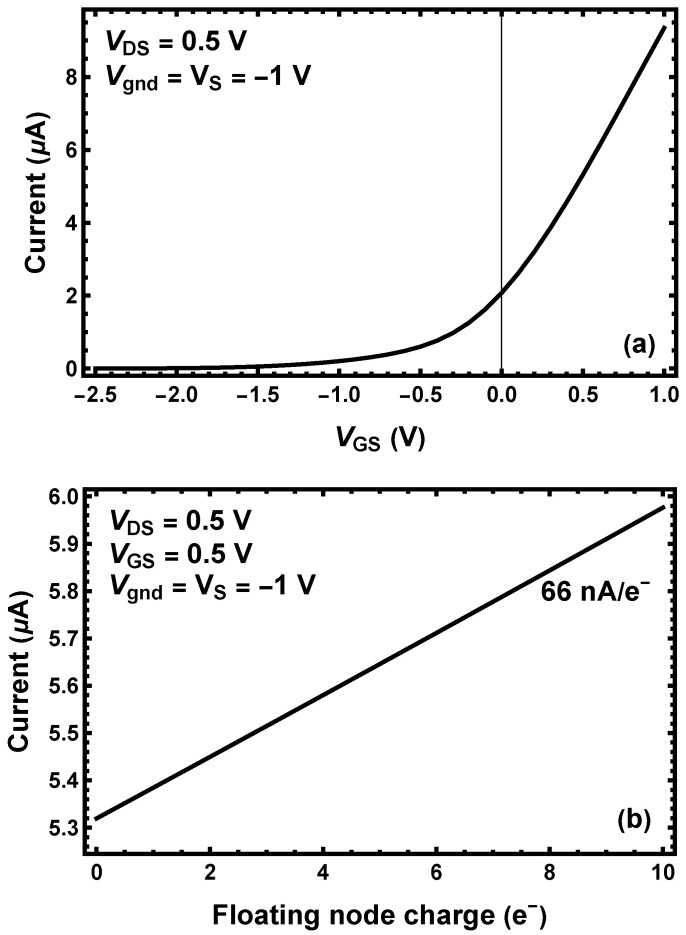
(**a**) The current-voltage characteristic of the MOSFET with the parameters shown as a function of the gate-source voltage. (**b**) The MOSFET current as a function of the number of electrons on the floating node. By combining the data in these two panels, we estimate that one additional electron on the floating node is equivalent to about 10 mV change in $V_{\mathrm{GS}}$.

The results of Comsol simulations are given in Figure 4. Figure 4a illustrates that the MOSFET considered here has a threshold as the gate voltage is raised above the source voltage. We pick a voltage on the transition between the regime of exponential and linear growth in current with an increase in the gate voltage as the operating point. In the example, $V_{\mathrm{DS}}=V_{\mathrm{GS}}=0.5$ V where “D” is for “drain”, “S” is for “source”, and “G” is for “gate”. The ground is denoted as “gnd”. It is set to −1 V so that the Comsol Semiconductor Initialization study predicts an equal concentration of electrons and holes far from the circuit when all the voltages are set to the same value. The floating node gate is also set at the MOSFET gate voltage of 0.5 V.

The number of electrons on the gate for a given number of electrons forced on the floating node is given in Table 2. The number of electrons is plausible in light of the simple estimate given in Appendix F. We argue heuristically that if there are of order 10 electrons on the gate defining the flow of charge in a MOSFET, it is plausible that a single electron nearby would have an observable effect. Table 2 illustrates that adding electrons to the floating node reduces the need to have electrons on the gate to maintain a given voltage. Incidentally, the fact that the number of electrons on the gate is exactly 10.00 with no electrons on the floating node is a numerical coincidence.

Figure 4b contains the main result of the paper. Each electron added to the floating node causes a change of 66 nA in the MOSFET current or just over 1%.

To estimate thermal noise, we use numbers from a report [33] of a device similar to the one we propose. When operated with a gate voltage of 0.5 V, the noise decreases from $3\times 10^{-11}$ A/Hz^1/2^ at 10 kHz to $4\times 10^{-12}$ A/Hz^1/2^ at 400 kHz, then achieves a plateau at higher frequencies. For simplicity, we will take 400 kHz as the operating frequency of our device. For a 2.5 μs observation, a noise current of 2.5 nA is expected. Since one electron leads to a current difference of 66 nA per electron (26 standard deviations), the MOSFET noise current is not a significant issue. We can operate with less observation time, namely 1.0 μm, with negligible electron number assignment error with an expected noise current $\sqrt{2.5}$ times higher, or 4.0 nA (1/17 of 66 nA). The relation of noise and observation time is given for a related device [32]). The timing cycle is shown in Figure 1c. The current of the MOSFET will be measured with a transimpedance amplifier. Commercially available high-precision transimpedance amplifiers have noise in the picoampere range which is negligible for our situation. Moreover, a 1 μs measurement time is achievable with routine, if careful, design. It takes about 1 ns to clear the photoelectrons. While the noise estimate is made at 300 K, cooling to 263 K should reduce the noise, although measured values are not available in the reference.

### 2.5. Device Reset

When the number of electrons in the floating node is being measured, the clearance gate is set to a low voltage, typically 1 V less than the floating node gate and the drain voltage. When the electrons in the floating node are to be cleared, the floating node is set to a low voltage (e.g., 0 V), the clearance gate to an intermediate voltage (e.g., 1 V), and the drain to a high voltage (e.g., 2 V). The gate on the MOSFET should also be set to a low voltage. Because the distance the electrons in the floating node have to travel is very small, much less than 1 μm, the clearance step is very fast, e.g., 1 ns or less. The electrons on the floating node and any electrons in the absorption or transport region are cleared in the 60 ns period alloted for clearance.

The holes are cleared continuously. The mobility of the holes is about three times less than the mobility of the electrons, so it could take more than one cycle to clear the holes. However, the device performance is nearly independent of the number of holes in the absorption region.

### 2.6. Carrier Lifetime Effects

**Figure 5:**
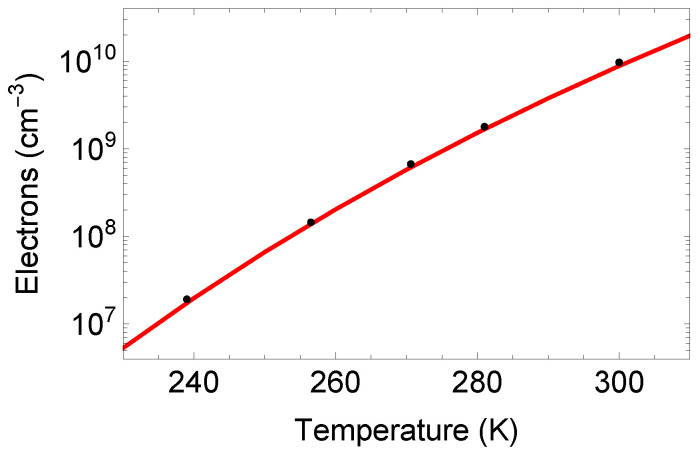
The electron carrier concentration in intrinsic silicon as measured [39] (black circles) and from a web-based calculator [40] (red line).

Conduction-band electrons can be created thermally. A review [34] and another study [35] of the creation and recombination of electron–hole pairs in silicon has appeared. The situation is rather complicated and depends on the material, so we quote instead a record reported carrier lifetime in silicon of 8.5 ms [36]. For our example, we pick 5 ms as the electron lifetime in room temperature silicon and derate that to 4 ms at 263 K [37]. The equilibrium intrinsic carrier concentration in silicon at 300 K is $1.0\times 10^{10}$ cm^−3^ [38]. This implies a carrier generation rate of $2.0\times 10^{12}/$(cm^3^·s). At 263 K, the intrinsic carrier concentration is much lower at $2.8\times 10^{8}$ cm^−3^ [39,40] leading to a carrier generation rate of $7\times 10^{10}/$(cm^3^·s). The electron carrier concentration in intrinsic silicon as a function of temperature is plotted in Figure 5.

Assuming a device volume of $1.5\;V_{\mathrm{abs}}=7\times 10^{-13}$ m^3^ and a time of 72 ns, at room temperature 0.2 carrier electrons would be generated during clearing and transport phases, although only half of these (those created late in the clearing phase or early in the transport phase) would reach the floating node during the transport phase, i.e., 0.1 electrons. This would represent a 10% error rate, which is not consistent with our design goals. At 263 K, the carrier generation rate is much lower and the time for clearing and transport is also reduced leading to an expected background of 0.003 electrons, which is acceptable. The reasoning in this section shows why we propose operating at −10 °C despite some complications in the implementation shown in Figure 2.

Electrons at the surface have a shorter lifetime than those in the bulk. Because the electrons spend a considerable fraction of their time in a thin layer near the silicon-silicon dioxide interface, this lifetime is a key parameter for our device. Larionova et al. report lifetimes of 2.4 ms ± 0.2 ms for an annealed interface with 10 nm of silicon dioxide and 2.1 ms ± 0.2 ms for a 40 nm thick layer [41]. Here, we propose an oxide layer 20 nm thick. We assume a lifetime of 2 ms for surface interactions. For convenience, we also use the 2 ms lifetime for the bulk although 4 ms can be justified from the literature. We recognize that careful material processing will be required to achieve this result.

### 2.7. Error Rate Estimate

Here, we simulate a device which is capable of resolving up to 10 visible light photons in a pulse with very small error rate. Specifically, the chance of an error by 1 in the photon number is 2.1%. To put it a different way, our quoted error rate describes the performance of a real device as compared to a hypothetical ideal device which would always report the correct actual number of photons incident on the entrance plane.

The calculation has been performed relative to the number of photons arriving in a pulse at the input face of the antireflective coating covering the absorption region. Due to the discrete nature of photons and electrons, the output voltages should be strongly peaked allowing for a relatively obvious classification of the output voltage ranges into discrete numbers of incident photons.

In Table 3, the probabilities are added linearly because they represent discrete, uncommon events. The signals are assumed to be rounded to a discrete integer number of photons. This requires a noise excursion in excess of 0.5/0.04=12.5 standard deviations for the short-channel MOSFET to cause a discretization error which is negligible.

The sources of error are collected in Table 3. Because the photon number is discrete, we ask what is the chance that the results will be low or high by 1 photon. For 1 incident photon with λ=700 nm, the results will be low 0.18% of the time and high 0.3% of the time. If there are 10 incident photons, there is 1.8% chance of being low while retaining a 0.3% chance of being high. With more incident photons, the chance of being low would exceed the chance of being high. Collectively, there is over a 99% chance of reporting the correct photon number with up to 10 incident photons in a pulse.

The parameters underlying Table 3 impacts the measurement outcome of a particular input pulse. By characterizing the device, we may ascertain with low uncertainties the reflectivity of the entrance plane and other parameters. However, this does not reduce the error rate of the measurement of the number of photons in a particular pulse.

Our proposed operating point is close to the maximum rate as determined by requiring a low-error electron-number measurement. The time is dominated by the measurement time which is already close to the minimum before electron assignment errors become significant. The greatest hope for improvement in efficiency would be to improve the optical capture rate to be closer to the theoretical limits for a Gaussian beam.

## 3. Conclusions

We have proposed a near-room temperature, MOS-based device which we estimate can be used as an accurate, primary standard for photon-number-resolving (PNR) detection. By “primary,” we mean a measurement which does not require additional external calibrations. We have shown that this proposed device would offer, at or near room-temperature operation, a PNR error rate competitive with detectors which must be run below 4.2 K. Cooling from room temperature to −10 °C can be achieved with thermoelectric coolers which are solid-state devices. Even though the device must be cooled, the package would be operated at room temperature.

We have shown theoretically and through simulation that a 263 K (−10 °C) silicon device is capable of resolving pulses with up to 10 photons with an estimated photon-number error rate of 2.1% with 900 kHz operation. The device can be fabricated with common techniques, although it does rely on thinning silicon to 30 μm and depositing both circuitry and optical coatings on both the top and bottom sides of the device. With some simplification, the principle of device operation is to convert photons to conduction-band electrons in a cylinder 125 μm in diameter and 30 μm thick. These photoelectrons are then moved toward a MOSFET. The presence of those nearby electrons leads to a variation in the current through the MOSFET which can be distinguished at the single electron level in about 1 μs. Using the same arguments given in the article, a device up to 50 μm in diameter would be capable of similar performance at room temperature (300 K) at the price of making the optical coupling more difficult.

While the device may be broadly applicable to calibrating photon-counting array detectors, one specific application would be to replace a cryogenically cooled transition-edge sensor with a room temperature package in a photonic quantum computer.

## Figures and Tables

**Table 1:** Temperature-dependent parameters used or derived in the estimate of the time to transport electrons from the absorption region to the floating node, minimum thermodynamically required voltages, and quantities required to estimate the error rate.

Temperature (K)	300	263
Mobility–bulk (cm^2^/(V·s))	1440	1800
Mobility–near interface (cm^2^/(V·s))	500	600
Drift velocity (m/s)	5500	6600
Drift time (ns)	72	60
Diffusion coefficient *D* (m^2^/s)	0.0039	0.0046
$V_{\min}$ (V)	0.69	0.60
$V_{\eta_{0}}$ (V)	0.99	0.86
Electron lifetime–bulk (ms)	5	4
Electron lifetime–near interface (ms)	2	2
Intrinsic carrier concentration (1/cm^3^)	$1.0\times 10^{10}$	$2.8\times 10^{8}$
Carrier generation rate (1/(cm^3^· s))	$2.0\times 10^{12}$	$7\times 10^{10}$

**Table 2:** The number of electrons on gates under the conditions of Figure 4.

Floating Node Electrons	Gate Electrons
10	10.00
10	19.61

**Table 3:** Uncertainty budget for the photon-number determination $N_{\mathrm{phot}}$ for the proposed device, assuming 1 or 10 incident photons. Some entries are proportional to the number of incident photons and others are independent of this value. Processes which lead to an increase, decrease, or either in the reported photon number are marked with the symbols +, −, and ±, respectively.

Issue	Sign of Effect	Uncertainty		Remark
		$N_{\mathrm{phot}}=1$	$N_{\mathrm{phot}}=10$	
Discrete photon-number uncertainties				
Reflection from three front surfaces	−	0.0002	0.002	AR coating (99%) with recycling mirror (99.4%)
Reflection loss, bottom mirror	−	0.0001	0.001	λ=700 nm, 0.33% to mirror, 3% absorbed
Transmission through absorption region	−	$2\times 10^{-7}$	$2\times 10^{-6}$	λ=700 nm (worst in visible)
Beam-aperture interaction Equation (1)	−	0.001	0.010	$w_{0}=18\;\mu$m, a=62.5μm
Subtotal		0.0013	0.013	Added linearly
Discrete electron number uncertainties				
Electrons generated in absorption region	+	0.003	0.003	Clearing before pulse, gate
Recombined electrons	−	0.0005	0.005	1.06 μs transport plus measurement time over 2 ms lifetime
Multiple photoelectron generation	+	negligible		λ≥400 nm
Lagging electrons	−	negligible		60 ns twice average transit time
Electrons that never reach floating node	−	negligible		
Subtotal		0.0035	0.008	Added linearly
Continuous uncertainties				
Short-channel MOSFET noise	±	negligible		0.06 photon equivalent standard error
Subtotal		negligible		Not likely to cause error after rounding
Total		0.005	0.021	Added linearly

## Data Availability

Data is available from the authors upon reasonable request.

## Supplementary Materials

The following supporting information can be downloaded at: https://www.mdpi.com/article/10.3390/s25175470/s1, Video S1: Electrons moving from absorption region to under the wedge electrode.

## Appendix A. Terms Related to “Error Rate”

We use the term “error rate” in this paper because it is recognized across many technical disciplines. It is equivalent to the “*n* photon infidelity” (i.e., one minus the “*n* photon fidelity”) used in the quantum measurement community [11] as well being one minus the “system detection efficiency” for *n* photons.

We reserve the term “uncertainty” for continuous quantities, although this practice is not universal. It may be natural to refer to an uncertainty of one or two photons in a distribution which has a mean of, say, 50 or 100, but we do not pursue the crossover from discrete to continuous distributions here.

## Appendix B. Poisson Distribution and Photon Uncertainty

Ideally, the detector, after some processing in software, will report a number of electrons detected $N_{d}$. Assume there are $N_{i}$ incident photons. The goal of the uncertainty analysis is to determine the distribution for $N_{i}$ given $N_{d}$. We may use Bayes’ Theorem to write

(A1) $$\begin{array}{c} P\left(N_{i}|N_{d}\right)\;P\left(N_{d}\right)=P\left(N_{d}|N_{i}\right)\;P\left(N_{i}\right). \end{array}$$

Let us assume that $P(N_{d})$ and $P(N_{i})$ are slowly varying functions which can be taken to be unknown constants. If *C* is their ratio,

(A2) $$\begin{array}{c} P\left(N_{i}|N_{d}\right)\approx C\;P\left(N_{d}|N_{i}\right). \end{array}$$

Assuming that the number of incident and detected photons is large and $N_{d}\leq N_{i}$, then letting $\mu =RN_{i}$ where R<<1 is the fractional loss of photons and μ is the mean of a Poisson distribution characterizing the number of lost photons, then

(A3) $$\begin{array}{c} P\left(N_{d}|N_{i}\right)=\frac{\mu^{Ni-Nd}e^{-\mu }}{(N_{i}-N_{d})!}. \end{array}$$

Next, we observe that the range of incident numbers of photons which are plausibly related to a detection of $N_{d}$ is from 0 to a few. Hence μ is approximately constant in the range of interest. If this constant is $\mu_{0}$, then

(A4) $$\begin{array}{c} P\left(N_{d}|N_{i}\right)\approx \frac{\mu_{0}^{Ni-Nd}e^{-\mu_{0}}}{(N_{i}-N_{d})!}. \end{array}$$

Hence, $N_{i}-N_{d}$ approximately follows a Poisson distribution with parameter $\mu_{0}$. To achieve our design goals, we require $\mu_{0}<\;<1$. More particularly, if we want an error rate of at most 0.05, then $\mu_{0}<0.05$, which follows by considering two adjacent probabilities given by Equation (A4) with $N_{i}-N_{d}=0$ and $N_{i}-N_{d}=1$ and neglecting a loss of more than one photon. If $N_{i}=10$, $R<\frac{\mu_{0}}{N_{i}}=0.005$. In practice, *R* should not be too close to 0.005 lest there is no source of error allowed for the rest of the device.

## Appendix C. Absorption with the Back Mirror

Equation (3) can be cast as a quadratic equation

(A5) $$\begin{array}{c} R_{M}X^{2}+A_{M}X-T=0 \end{array}$$

where X=exp(−αL) and A=1−T is the required absorption, $R_{M}$ is the reflectivity of the mirror, and $A_{M}=1-R_{M}$. Solving the quadratic and selecting the positive root leads to the solution

(A6) $$\begin{array}{c} \alpha =\frac{1}{L}\ln\left[\frac{2R_{M}}{{\left(A_{M}^{2}+4TR_{M}\right)}^{1/2}-A_{M}}\right]. \end{array}$$

## Appendix D. Least-Time Theorem

Suppose an electron is moving subject to drift only, i.e., not diffusion. Here, we consider which set of electric fields will transport a particle along a 1D path in the shortest time subject to a specified total voltage difference V>0 with a continuous electric field. The electron velocity is given by

(A7) $$\begin{array}{c} v=-\mu E. \end{array}$$

Here, a scalar form is adopted as we consider a one-dimensional path parameterized by a coordinate ξ with dimensions of length from $\xi_{0}$ to $\xi_{1}$ with $L=\xi_{1}-\xi_{0}$. The time *t* it takes to traverse the path is

(A8) $$\begin{array}{c} t=\int_{\xi_{0}}^{\xi_{1}}\;d\xi \;\frac{1}{v(\xi )}=-\frac{1}{\mu }\int_{\xi_{0}}^{\xi_{1}}\;d\xi \;\frac{1}{E(\xi )}. \end{array}$$

Let $E\left(\xi \right)=E_{0}+E_{1}\left(\xi \right)$ with $E_{0}=-V/L$ and $E_{1}\left(\xi \right)$ is a continuous function. As a corollary,

(A9) $$\begin{array}{c} \int_{\xi_{0}}^{\xi_{1}}\;d\xi \;E_{1}\left(\xi \right)=0. \end{array}$$

We may write

(A10) $$\begin{array}{ccc} t & = & -\frac{1}{\mu E_{0}}\int_{\xi_{0}}^{\xi_{1}}\;d\xi \;\frac{1}{1+E_{1}\left(\xi \right)/E_{0}}\\ & = & -\frac{1}{\mu E_{0}}\int_{\xi_{0}}^{\xi_{1}}\;d\xi \;\left(1-\frac{E_{1}\left(\xi \right)}{E_{0}}+\frac{E_{1}^{2}\left(\xi \right)}{E_{0}^{2}}\frac{1}{1+E_{1}\left(\xi \right)/E_{0}}\right)\\ & = & -\frac{1}{\mu E_{0}}\int_{\xi_{0}}^{\xi_{1}}\;d\xi \;\left(1+\frac{E_{1}^{2}\left(\xi \right)}{E_{0}^{2}}\frac{1}{1+E_{1}\left(\xi \right)/E_{0}}\right)\\ & = & \frac{L^{2}}{\mu \;V}+\frac{1}{\mu }\int_{\xi_{0}}^{\xi_{1}}\;d\xi \;\frac{E_{1}^{2}\left(\xi \right)}{E_{0}^{2}}\frac{1}{\left[-E(\xi )\right]}. \end{array}$$

The final term is nonnegative because E(ξ)≤0 everywhere. If it were positive, then v<0 and then electron would never get from $\xi_{0}$ to $\xi_{1}$. Hence, the least time path has $E_{1}\left(\xi \right)=0$. Physically, the least time path is a constant electric field. The proof does not require $E_{1}\left(\xi \right)/E_{0}<\;<1$. In the case that if the mobility varies with position, i.e., μ=μ(ξ), a similar argument shows that if the mobility varies with position, μ(ξ)E(ξ) is constant on the least-time path.

## Appendix E. The Laplace Equation and Wire Spacing

Ideally, we have a potential which is a linear function of position *x* at the silicon-oxide surface at $y=y_{0}$. However, typically, the potential is imposed by wires on top of the oxide surface which have a space between wires equal to the wire width *w*. Hence, *w* is the half pitch. To have a simple model, the potential at the interface of the wires and the oxide, the $y=y_{0}$ plane, obeys

(A11) $$\begin{array}{c} \phi \left(y_{0},z\right)=-\frac{V_{w}}{4}+\frac{V_{w}}{2}\frac{z}{w}+\frac{V_{w}}{4}\cos\frac{\pi z}{w}, \end{array}$$

where $V_{w}$ is the voltage difference between adjacent wires. This form comes from fitting a constant field plus a triangle wave, keeping the fundamental Fourier coefficient, and matching the voltages to at the edges of the wires. With this boundary condition, the potential in the oxide obeys

(A12) $$\begin{array}{c} \phi \left(y,z\right)=-\frac{V_{w}}{4}+\frac{V_{w}}{2}\frac{z}{w}+\frac{V_{w}}{4}\cos\frac{\pi z}{w}e^{\kappa (y-y_{0})} \end{array}$$

with $\kappa =\frac{\pi }{w}$. If we made a more rigorous representation of a triangle wave potential, the Fourier components with wave vectors larger than $\frac{\pi }{w}$ would fall even faster away from the wiring plane, so we neglect these.

If we want the electrons to move forward based on drift (i.e., as opposed to relying on diffusion which is slower), then we need ϕ(0,z) to increase with *z*, taking y=0 at the semiconductor-oxide plane, then we require

(A13) $$\begin{array}{c} \frac{d}{dz}\phi \left(0,z\right)=\frac{V_{w}}{2w}+\frac{\pi V_{w}}{4w}\sin\frac{\pi z}{w}e^{-\kappa y_{0}}>0. \end{array}$$

Taking *z* so that the sin function is −1,

(A14) $$\begin{array}{c} w<\frac{\pi }{\ln(\pi /2)}y_{0}\approx 7y_{0}. \end{array}$$

Typical values for the wire width *w* and the oxide thickness $y_{0}$ are 25 nm and 20 nm, so we expect this condition to be relatively easy to achieve. In light of Appendix D, the wire width *w* should be as small as practical, since this will lead to a nearly constant electric field.

## Appendix F. Parallel-Plate Capacitance

We can estimate the numbers of electrons on the MOSFET gate from the parallel plate capacitance formula

(A15) $$\begin{array}{c} C=\frac{\epsilon A}{d}, \end{array}$$

where $\epsilon =\epsilon_{R}\;\epsilon_{0}$ is the dielectric constant, *A* is the area of the capacitor plate, and *d* is the plate thickness. Here, $\epsilon_{R}$ is the relative dielectric constant and $\epsilon_{0}$ is the permittivity. For a simple approximation, we model the device as a parallel plate capacitor without fringing with the two plates being the MOSFET gate and the MOSFET channel with an oxide layer in between.

If we take the silica dielectric constant to be $\epsilon_{R}=3.5$, A=5000 nm^2^, and d=20 nm, then C=7.7 aF. Using the defining relation for capacitance C=Q/V, where *Q* is the charge on one plate of a capacitor and *V* is the voltage. Taking V=0.5 V as an example, there would be 24 electrons on the capacitor. This is a factor of 2 or 3 greater than the values given in Table 2.
